# Beyond the cross-sectional snapshot: longitudinal trends, regional disparities, and the pandemic-related disruption of spirometry in Brazil

**DOI:** 10.36416/1806-3756/e20260202

**Published:** 2026-09-14

**Authors:** Marcos Brum-Antunes, Alícia Viana Tavares, Jordana Henz Hammes, Marcia Margaret Menezes Pizzichini, Marcus Herbert Jones

**Affiliations:** 1. Programa de Pós-Graduação em Pediatria e Saúde da Criança, Escola de Medicina, Pontifícia Universidade Católica do Rio Grande do Sul - PUCRS - Porto Alegre (RS) Brasil.; 2. Programa de Pós-Graduação em Ciências Médicas, Universidade Federal de Santa Catarina - UFSC - Florianópolis (SC) Brasil.

## TO THE EDITOR:

Our recent article showed that spirometry records in the Brazilian public health care system reveal substantial disparities in the distribution of testing across the country, with a higher concentration in areas served by better-structured healthcare networks.^1^ The study provided a robust contemporary overview of the spirometry registry in Brazil, showing that access to the test remains far from equitable. However, this cross-sectional snapshot does not allow assessment of the trajectory of spirometry provision.^2^ ^-^ ^4^

It is precisely in this context that a longitudinal approach becomes necessary. This analytical extension is particularly relevant to understanding the historical evolution of access to spirometry, since the availability of the test depends on installed capacity, regular outpatient care pathways, trained personnel, and operational conditions that have been substantially strained over time, especially during the COVID-19 pandemic.^5^

Procedure records from the Brazilian public health system were analyzed using outpatient production data obtained from the Brazilian Health Informatics Department of the Ministry of Health.^6^ ^,^ ^7^ Rates were calculated using population denominators derived from the Automatic Retrieval System of the Brazilian Institute of Geography and Statistics, with a coverage adjustment based on subtracting 25% of the total population as an approximation of the proportion covered by private health insurance and therefore less reliant on public provision of spirometry.^8^

To assess the effect of the pandemic, an interrupted time-series approach was applied, with a 2020 segmentation point to estimate the pre-pandemic trend, the immediate change in level at the onset of the pandemic, and the subsequent change in slope.

Between 2010 and 2024, a total of 3,406,788 spirometry tests were recorded in Brazil. At the national level, spirometry provision expanded steadily during the pre-pandemic period, with the number of tests increasing by 80.0% from 2010 to 2019. This trajectory was abruptly interrupted in 2020, followed by substantial recovery in subsequent years. By 2024, the number of spirometry tests was 125.9% higher than in 2010 and 189.9% higher than in 2020 (Figure 1).

**Figure 1:**
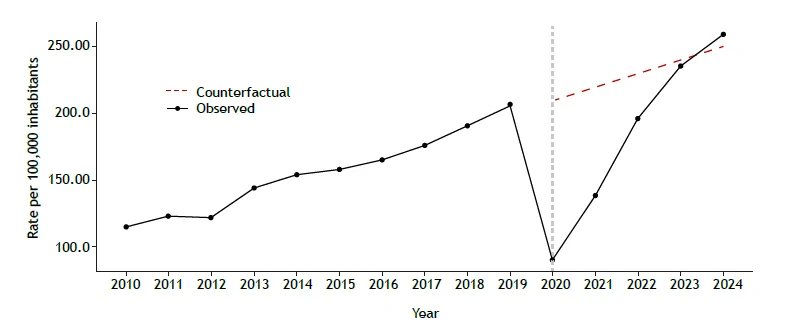
Observed and counterfactual annual spirometry rates in Brazil (2010-2024). The black solid line represents the observed rate per 100,000 population, and the red dashed line represents the counterfactual rate projected from the pre-pandemic trend. The vertical dotted line marks the onset of the COVID-19 pandemic in 2020.

This expansion, however, was uneven across the country (Figure 2). Between 2010 and 2024, spirometry volume increased in all macroregions, with the largest relative growth observed in the South (417.0%), followed by the Northeast (155.3%), Southeast (99.2%), North (77.5%), and Central-West (76.7%). During the pre-pandemic period alone, growth was already evident in most regions, particularly in the South (287.6%) and Northeast (89.5%), whereas the North showed a 26.0% decline between 2010 and 2019. These findings suggest that the national upward trend reflected not a uniform expansion of access but rather heterogeneous regional trajectories.

**Figure 2:**
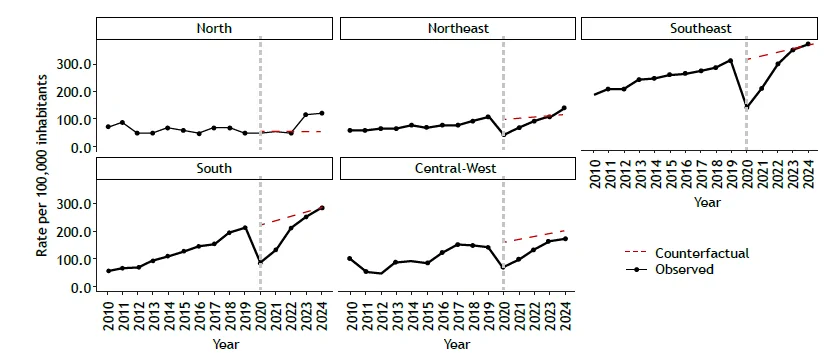
Observed and counterfactual annual spirometry rates by Brazilian macroregion (2010-2024). Black solid lines represent observed rates per 100,000 population and red dashed lines represent counterfactual rates projected from the pre-pandemic trend. The vertical dotted line marks the onset of the COVID-19 pandemic in 2020.

Territorial coverage remained limited throughout the series. In each year, spirometry was offered in only 130-362 of Brazil’s 5,570 municipalities, corresponding to 2.3% in 2010 and 6.5% in 2024. Over the full study period, only 487 municipalities recorded at least one spirometry test, representing 8.7% of all municipalities in the country. Thus, despite temporal increase in test volume, spirometry provision remained geographically concentrated.

Taken together, these findings suggest a gradual expansion in diagnostic capacity within the Brazilian public health care system before the pandemic.^6^ ^,^ ^9^ Nevertheless, the level of provision remained low relative to the likely burden of respiratory disease in the population. In 2024, the observed spirometry rate in Brazil reached 257.98 per 100,000 population, and the national median rate during 2020-2024 was 194.8 per 100,000. By contrast, the estimated prevalence of asthma among adults is approximately 4,600 per 100,000. Although these measures are not directly comparable, this contrast highlights a substantial gap between diagnostic provision and potential population need for respiratory care. Even under a conservative scenario based on the average annual growth observed over the full 2010-2024 period, and under the simplifying assumption of one spirometry test per adult with asthma, Brazil would still require approximately 50 years to reach a spirometry rate of the same order of magnitude as the estimated prevalence of adult asthma.^10^ ^,^ ^11^

Against this background, the COVID-19 pandemic caused a clear disruption in the national trajectory. In Brazil, observed spirometry provision during 2020-2024 remained 14.7% below the counterfactual level, with a mean shortfall of 21.5% and an interquartile range from −37.3% to −1.8%. The model also identified a marked immediate decline in 2020 of 70.3% (95% CI: −79.8% to −60.8%; p < 0.001), consistent with an abrupt interruption in test provision at the onset of the pandemic. This pattern is coherent with the suspension of elective procedures, the reorganization of care pathways, operational restrictions within health services, and the postponement of respiratory function testing in a context in which spirometry itself came to be viewed as a higher-risk procedure because of aerosol generation.

Although a significant positive change in the post-pandemic slope was observed in Brazil (+16.1%; 95% CI: 13.5% to 18.7%; p < 0.001), recovery remained insufficient to restore the level expected in the absence of the pandemic. Regionally, the largest relative deficits compared with the counterfactual were observed in the Central-West (−26.2%), South (−17.0%), Northeast (−15.5%), and Southeast (−12.9%), whereas the North showed little overall deviation (−0.3%). All regions experienced a significant immediate decline in 2020, ranging from −65.7% in the Central-West to −85.3% in the North. Post-pandemic slope increases were also significant across all macroregions, with the fastest recovery estimated for the North (+43.0%), followed by the Northeast (+19.7%), South (+15.5%), Southeast (+15.1%), and Central-West (+11.0%).

The regional disparities in spirometry provision within the Brazilian public health care system should be interpreted in the context of a historical trajectory of unequal expansion that was abruptly interrupted by the COVID-19 pandemic and only partially restored in subsequent years. Monitoring the recovery of service provision and reducing these regional asymmetries should remain a priority for the organization of respiratory care within the Brazilian public health care system.
